# Characterizing multidimensional worst-case scenarios in elite youth basketball: effects of time window and playing position

**DOI:** 10.3389/fspor.2026.1895096

**Published:** 2026-08-21

**Authors:** Yannis Irid, Julian Hutin, Jean-François Toussaint, Adrien Sedeaud

**Affiliations:** 1 IRMES-UMR 7329, Institut de Recherche Médicale et D’Épidémiologie du Sport, Université Paris Cité, Institut National du Sport, de L’Expertise et de la Performance (INSEP), Paris, France; 2 Fédération Française de Basketball, Paris, France; 3 Centre D’Investigation en Médecine du Sport, Assistance Publique—Hôpitaux de Paris, Paris, France

**Keywords:** basketball, external load monitoring, local positioning system, peak demands, worst-case scenarios

## Abstract

This study investigated multidimensional worst-case scenarios (WCS) in elite youth male and female basketball players. Data were collected from 31 players (male: *n* = 20; female: *n* = 11) competing at the French National Institute of Sport across 40 official matches over two consecutive seasons. Player activity was monitored using a 20-Hz Local Positioning System and 100-Hz embedded accelerometers. For each player, match, and time window (10, 30, 60, and 120 s), eight external load variables were extracted using rolling-window analyses. Variables were subsequently normalized and combined into a composite score, with WCS defined as the rolling window exhibiting the greatest simultaneous accumulation of external load demands. Linear mixed models were used to examine the effects of time window and playing position (frontcourt vs. backcourt) on WCS characteristics. Across both male and female players, shorter time windows consistently produced greater relative physical demands than longer epochs for most variables. However, jumps and changes of direction demonstrated fewer differences across time windows than locomotor variables. Position-specific differences were also observed, particularly among male players, with backcourt players generally exhibiting greater locomotor demands than frontcourt players during WCS. In female players, positional differences were less pronounced and primarily limited to selected acceleration-, deceleration-, and sprint-related variables. These findings suggest that the most physically demanding passages of basketball competition are highly dependent on the temporal scale considered and vary according to playing position. More importantly, the multidimensional WCS approach highlights periods characterized by the greatest concurrent accumulation of external load demands, providing a more holistic representation of match demands than traditional peak-demand analyses. These findings may assist practitioners in developing more specific training strategies to prepare athletes for the most challenging passages of play encountered during competition.

## 1 Introduction

In recent years, the focus on match and training load monitoring in basketball has intensified, leading to a surge in studies analyzing the most demanding scenarios (MDS) encountered by players during training or competition (1). These scenarios are critical for understanding the physical demands placed on athletes and for optimizing their performance (2, 3). In basketball, a MDS is defined as a period characterized by maximal or near-maximal physical demands that exceed 80% of a player's maximum intensity within a specified timeframe (4). These scenarios can be further subdivided into Peak Demand (PD), referring to the highest intensity activity for a particular variable, High Intensity Periods (HIP) and Very High Intensity Periods (VHIP), defined as intensity levels covering 80%–90% and >90% of PD, respectively (1, 4). The importance of accurately identifying and analyzing these periods is underscored by the limitations of relying on average values alone, as these often underestimate the true intensity of critical periods and overlook the intermittent nature of the game (5, 6). Consequently, moving averages have been proposed as one of the most suitable methods for capturing PD, offering a more precise understanding of the physical challenges athletes face (7).

The concept of WCS has recently emerged as a promising framework for capturing the most physically demanding passages of play in team sports. While PD provide valuable information regarding the highest intensity reached for individual variables, this approach remains largely univariate and may therefore fail to represent the multidimensional nature of match-play demands. In basketball, players are rarely exposed to maximal values of a single variable in isolation. Instead, the most challenging passages of play often involve the simultaneous accumulation of multiple locomotor and neuromuscular demands, including high running intensities, accelerations, decelerations, jumps, and changes of direction.

Consequently, a period characterized by the highest value for one specific variable does not necessarily coincide with the highest values of other performance indicators. This limitation may lead practitioners to underestimate the complexity and density of the most physically demanding passages encountered during competition. To address this issue, recent research has proposed the concept of WCS, defined as periods characterized by the greatest simultaneous accumulation of multiple external load demands (4). Rather than representing isolated maxima, WCS aim to identify passages of play where multiple physical demands are concurrently elevated, providing a more holistic and ecologically valid representation of competitive demands.

Although the WCS concept has received growing attention in team sports and has been explored in disciplines such as rugby, where collision and combat activities substantially contribute to overall load (8), its application in basketball remains scarce (1). Consequently, further research is required to better understand how these multidimensional scenarios manifest across different playing positions and time windows in basketball competition.

Despite the critical importance of these scenarios, there exists a research gap in analysis of WCS in basketball (1). To date, studies have focused on isolated physical metrics without examining the multifaceted nature of gameplay that WCS aim to capture (5, 9–16). Furthermore, although research on MDS in male basketball is extensive, there remains a notable gap concerning female athletes’ physical demands (4). This oversight is important, given the physiological and metabolic differences between male and female players, which may influence how these scenarios manifest in competition (4, 17). Addressing this gap is essential for developing tailored training strategies that enhance performance.

Therefore, the aim of this study was to characterize multidimensional WCS in elite youth basketball players and investigate the influence of time window duration, sex, and playing position on the physical demands experienced during these scenarios. Based on previous literature, we hypothesized that (i) shorter time windows would produce greater relative external load demands than longer time windows, and (ii) backcourt players would exhibit greater locomotor demands than frontcourt players during multidimensional WCS.

## 2 Materials and methods

### 2.1 Subjects

Data were collected from 31 elite youth basketball players (Male: *n* = 20; height: 199.83 ± 5.16 cm; mass: 86.13 ± 10.13 kg; age: 17.25 ± 0.62 years; Female: *n* = 11; height: 182 ± 8 cm; mass: 72.25 ± 11.08 kg; age: 17.42 ± 0.67 years) from the French National Institute of Sport (INSEP) teams over two consecutive seasons (2022-2023 and 2023-2024) during 40 official matches (males: *n* = 27; females: *n* = 13), resulting in 683 player-match samples (males: *n* = 540; females: *n* = 143). INSEP annually selects the top 50 French prospects (25 males, 25 females) aged 15–18, providing a high-performance environment aligned with recognized criteria for defining elite athletes (18, 19). These players represent France in U16, U17 and U18 international competitions and compete in national professional leagues [males in NM1 [3rd Division], females in LF2 [2nd Division]] to accelerate their development at the senior competitive levels. Throughout the data collection period, players trained approximately 15.5 h/week, comprising seven basketball sessions (technical, tactical, and competitive practice) and five strength and conditioning sessions. Participants were categorized into two positional groups—Backcourt (Point Guards, Shooting Guards; males: *n* = 11, females: *n* = 4) and Frontcourt (Small Forwards, Power Forwards and Centers; males: *n* = 9, females: *n* = 7)—to reflect the evolving, position-specific demands of modern basketball (20).

#### 2.1.1 Ethics statement

The collection of this data complies with the General Data Protection Regulations (GDPR) established by the European Union. The study was supervised and developed by the IRMES scientific committee. Ethical approval for the study protocol was obtained from the ethics panel of the Scientific, Medical, and Training Council (CSMF) at INSEP.

### 2.2 Data Collection

Players’ activities during every home game were recorded using a 20-Hz Local Positioning System (LPS) and 100-Hz embedded accelerometer devices (Kinexon, Kinexon GMBH, Munich, Germany) (21). The Kinexon Ultrawide Band (UWB) system consisted of 14 antennas positioned around the basketball court at the same heights, with players wearing tags on the upper back using manufacturer-provided harnesses. Player positions were calculated using proprietary algorithms based on Time Difference of Arrival, Two-Way Ranging, and Angle of Arrival methods (21). The validity of the Kinexon LPS device has been established, showing a small standardized typical error of estimate (from 0.06 to 0.48) compared with 3-dimensional motion capture (Vicon) for sprint, lateral, and specific handball movements (21, 22). Additionally, the interunit coefficient of variation ranged from 2.1 to 9.2% (22). All official matches were played on the same court under similar environmental conditions. Players were continuously monitored during each match, with data analysis limited to active play periods, excluding rest intervals and substitutions. Due to the permanent installation of the monitoring system at the INSEP facility, data from away official matches were not included. Only data from players who participated for at least three minutes of live play within the same quarter were included to mitigate performance variations associated with limited playing time (23, 24). The eight physical variables selected to describe match activities were: total distance covered (m), time spent running at high intensity (>18 km·h⁻^1^) (25), the number of accelerations and decelerations (>2 m·s⁻^2^), distance covered during high-intensity accelerations (>2 m·s⁻^2^) and decelerations (<−2 m·s⁻^2^), the number of high-intensity jumps (>0.40 m), and the number of high-intensity changes of direction (>90° combined with a deceleration <−2 m·s⁻^2^). These variables were selected because they represent complementary dimensions of basketball external load, including locomotor activity (total distance and high-speed running), acceleration-based demands (accelerations, decelerations, and their associated distances), and explosive basketball-specific actions (jumps and changes of direction). Collectively, these variables provide a comprehensive representation of the multidimensional physical demands encountered during basketball competition and are commonly used in basketball load-monitoring research (4, 5, 16). The selected thresholds were adopted from previous basketball literature using the Kinexon local positioning system to facilitate comparisons across studies (4, 25). The combined angular (>90°) and deceleration (<−2 m·s⁻^2^) criteria for changes of direction were used to identify only high-intensity directional changes while excluding low-intensity directional adjustments frequently occurring during match play.

### 2.3 Data processing

For each player, match, and time window (10, 30, 60, and 120 s), rolling-window analyses were performed using a 1-second step size. The selected time windows were chosen based on previous basketball research investigating MDS and have been identified as the most practical to consider in basketball (1). For every valid rolling window, eight external load variables were calculated: total distance covered, number of accelerations, acceleration distance, number of decelerations, deceleration distance, high-speed running duration, number of high-intensity jumps, and number of changes of direction.

Only valid windows recorded during official playing periods were retained for analysis. To ensure data quality, windows containing less than 50% valid tracking data were excluded. This threshold was selected to ensure that retained rolling windows contained sufficient valid tracking information while minimizing the exclusion of representative passages of play. Additionally, windows presenting a mean velocity lower than 0.5 m·s⁻^1^ were discarded to remove quasi-static periods of play (e.g., free throws, stoppages, or other low-movement phases) that were not considered representative of meaningful external load demands. Rolling-window analyses were reset at the end of each quarter and resumed at the beginning of the subsequent quarter.

For each variable and time window, PD was defined as the highest value observed across all valid rolling windows during the match. Subsequently, all variables were normalized using a min-max scaling procedure applied within each match, transforming each metric to a common scale ranging from 0 to 1. Min-max scaling was selected because it preserves the relative contribution of each variable while expressing all metrics on a common dimensionless scale, thereby allowing variables measured in different units to be combined into a single composite score.

**Figure 1:**
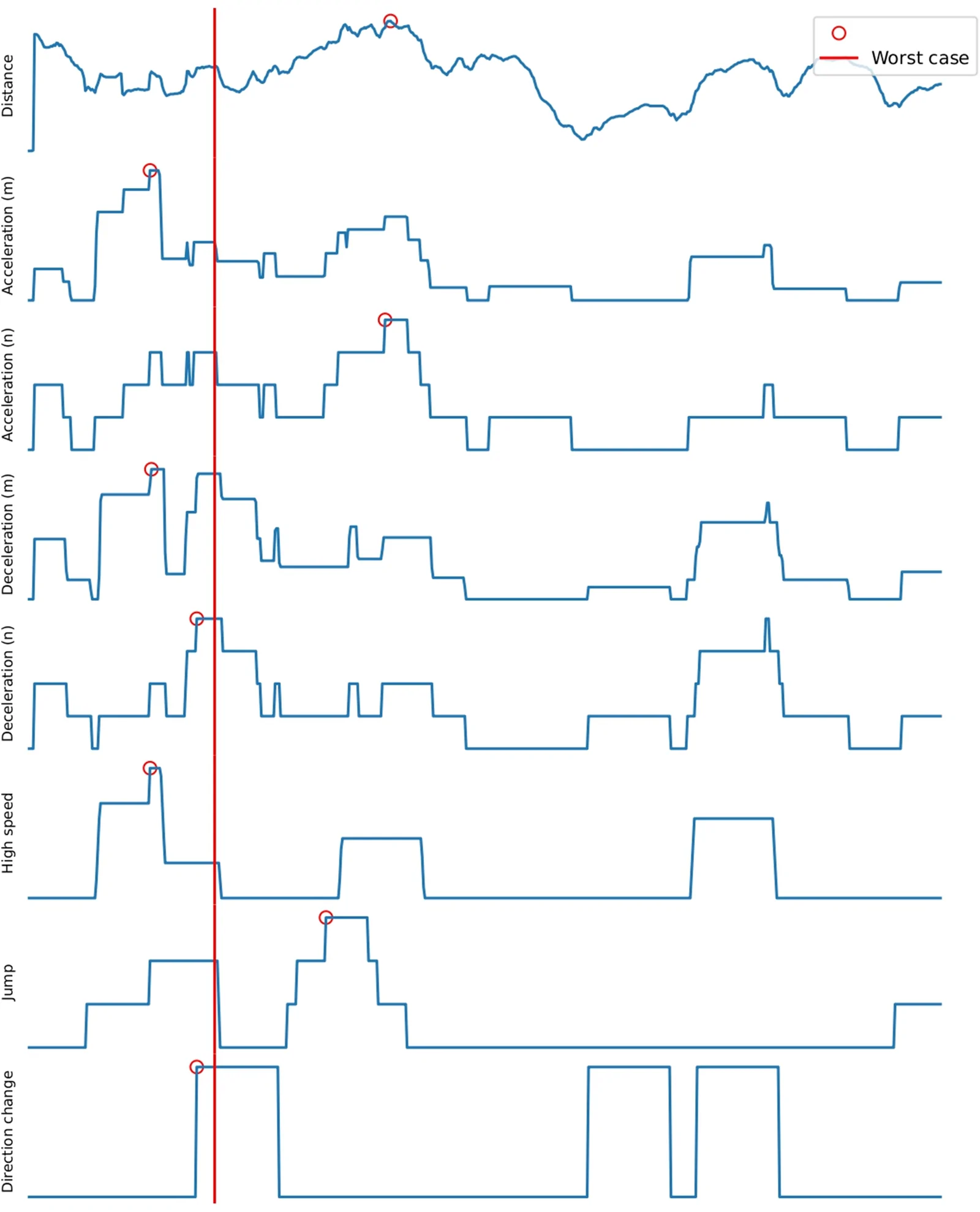
Identification of peak demands and worst-case scenarios across multiple physical variables. Identification of Peak Demands (PD) and Worst-Case Scenarios (WCS) across multiple external load variables. PD correspond to the highest value reached for each individual variable independently, whereas the WCS is defined as the rolling window exhibiting the highest composite score obtained from the simultaneous accumulation of all normalized external load variables. This framework illustrates the conceptual distinction between isolated peak values and multidimensional periods of maximal overall physical demand.

**Figure 2:**
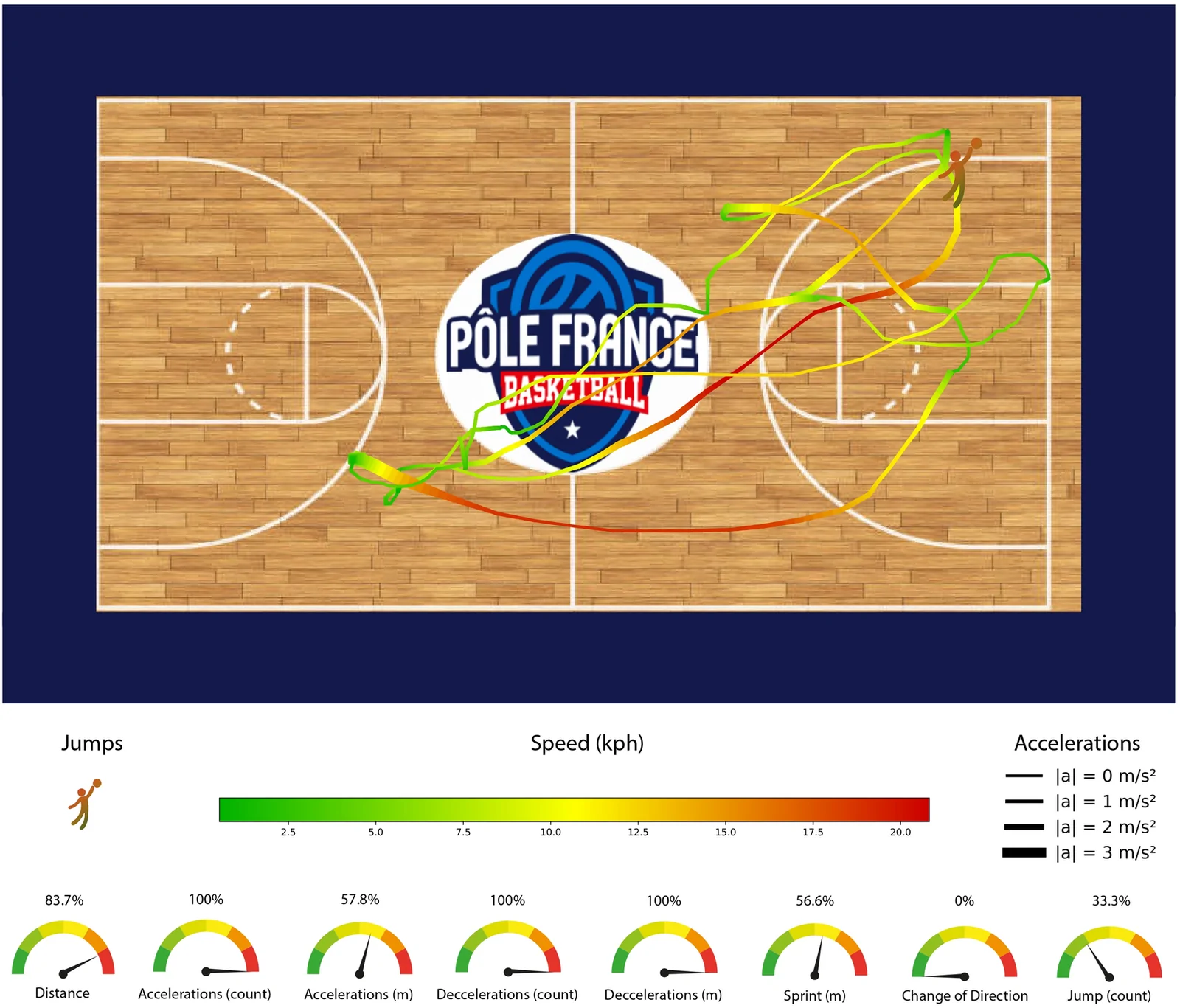
Graphical representation of a worst-case scenario over a 60-second period for One player. Example of a multidimensional Worst-Case Scenario (WCS) identified over a 60-second rolling window for one representative player. The highlighted period corresponds to the rolling window with the highest composite score, reflecting the greatest simultaneous accumulation of the eight external load variables included in the analysis. This example illustrates how the multidimensional approach identifies periods of elevated overall physical demand rather than isolated peaks in individual variables.

To identify the WCS, a composite score was calculated for every rolling window by summing the eight normalized external load variables. All variables contributed equally to the final score, with no weighting applied. Equal weighting was adopted to provide a transparent and reproducible framework for the initial exploration of multidimensional worst-case scenarios while avoiding subjective assumptions regarding the relative importance of each external load variable. This approach assumes an equivalent contribution of all variables to the composite score, which may not fully reflect their respective physiological significance. Consequently, the proposed framework should be considered as an initial methodological approach rather than a definitive weighting strategy. The WCS was then defined as the rolling window exhibiting the highest composite score throughout the match, representing the period characterized by the greatest simultaneous accumulation of external load demands across all selected variables (Figures 1, 2). The composite score was used exclusively to identify the rolling window corresponding to the multidimensional WCS and was not considered an outcome variable for subsequent statistical analyses, as it represents an arbitrary normalized index without direct physiological interpretation.

All physical variables during each identified WCS, together with the corresponding player coordinates, were subsequently extracted for further analyses.

### 2.4 Statistical analysis

Data are presented as mean ± standard deviation (SD). Prior to conducting the analyses, the normality of residuals was assessed using the Shapiro–Wilk test to ensure that assumptions for linear mixed modeling were met. To compare WCS between different time windows, variables were expressed relative to the window duration (in minutes). Linear mixed models were then employed to compare the WCS between different time epochs (10s, 30s, 60s, and 120s). In the model, time epoch was included as the fixed term (4 levels) and matches were included as a random term to account for multiple data samples obtained from several different matches. Matches were included as a random effect to account for repeated observations collected across multiple games. This modelling strategy was deliberately selected to address the predefined study objectives while maintaining model parsimony. Linear mixed models were also used to compare the WCS between playing positions in both male and female players, with position as the fixed effect (2 levels) and matches as a random effect.

All statistical analyses were performed using Python (Version 3.12.5), leveraging the ′statsmodels′ package for linear mixed models and ′scipy′ for normality testing. Effect sizes (ES) (Cohen's d) were calculated to provide a clearer interpretation of the magnitude of observed differences. The ES were interpreted as follows: ≤0.2, trivial; >0.2, small; >0.6, moderate; >1.2, large; >2.0, very large; and >4.0, nearly perfect (26). Statistical significance was set at *p* < 0.05.

## 3 Results

### 3.1 Time epoch impact

Significant differences were observed across all time epochs for both male and female basketball players in each WCS, except for jumps and changes of direction (Table 1). For male players, no significant differences were detected between the 60-second epoch and the 30-second and 120-second epochs in the number of jumps per minute (0.64 ± 0.81 n·min^−1^ vs. 0.96 ± 1.26 n·min^−1^; *p* = 0.062; d = 0.303, and 0.37 ± 0.42 n·min^−1^; *p* = 0.116; d = 0.419, respectively) or in the number of changes of direction between the 60-second and 120-second epochs (1.23 ± 0.91 n·min^−1^ vs. 0.76 ± 0.51 n·min^−1^; *p* = 0.075; d = 0.630).

**Table 1:** Average WCS during different time window for male and female teams. Player Position Differences.

Team	Variable	Window				*p*-values						Effect Size					
		10s	30s	60s	120s	*p* (10s-30s)	*p* (10s-60s)	*p* (10s-120s)	*p* (30s-60s)	*p* (30s-120s)	*p* (60s-120s)	d (10s-30s)	d (10s-60s)	d (10s-120s)	d (30s-60s)	d (30s-120s)	d (60s-120s)
Male	Distance (m.min-1)	208.8 ± 40.8^b^^,^^c^^,^^d^	152.74 ± 24.08^a^^,^^c^^,^^d^	123.36 ± 20.72^a^^,^^b^^,^^d^	97.42 ± 19.67^a^^,^^b^^,^^c^	< 0.001	< 0.001	< 0.001	< 0.001	< 0.001	< 0.001	1.674	2.641	3.478	1.308	2.516	1.284
	Accelerations (n.min-1)	10.56 ± 3.48^b^^,^^c^^,^^d^	5.5 ± 1.84^a^^,^^c^^,^^d^	3.74 ± 1.28^a^^,^^b^^,^^d^	2.54 ± 0.91^a^^,^^b^^,^^c^	< 0.001	< 0.001	< 0.001	< 0.001	< 0.001	< 0.001	1.805	2.585	3.132	1.115	2.043	1.079
	Accelerations (m.min-1)	30.6 ± 10.44^b^^,^^c^^,^^d^	14.14 ± 4.94^a^^,^^c^^,^^d^	8.93 ± 3.23^a^^,^^b^^,^^d^	5.96 ± 2.12^a^^,^^b^^,^^c^	< 0.001	< 0.001	< 0.001	< 0.001	< 0.001	< 0.001	2.012	2.799	3.265	1.246	2.151	1.087
	Decelerations (n.min-1)	10.32 ± 3.12^b^^,^^c^^,^^d^	5.38 ± 1.7^a^^,^^c^^,^^d^	3.66 ± 1.2^a^^,^^b^^,^^d^	2.47 ± 0.85^a^^,^^b^^,^^c^	< 0.001	< 0.001	< 0.001	< 0.001	< 0.001	< 0.001	1.963	2.806	3.418	1.158	2.149	1.140
	Decelerations (m.min-1)	31.86 ± 10.8^b^^,^^c^^,^^d^	14.7 ± 4.7^a^^,^^c^^,^^d^	9.08 ± 3.25^a^^,^^b^^,^^d^	5.92 ± 2.09^a^^,^^b^^,^^c^	< 0.001	< 0.001	< 0.001	< 0.001	< 0.001	< 0.001	2.059	2.856	3.333	1.391	2.411	1.152
	Sprint duration (s.min-1)	15.54 ± 7.98^b^^,^^c^^,^^d^	6.4 ± 3.56^a^^,^^c^^,^^d^	3.5 ± 2.1^a^^,^^b^^,^^d^	2.12 ± 1.13^a^^,^^b^^,^^c^	< 0.001	< 0.001	< 0.001	< 0.001	0.005	0.005	1.477	2.060	2.350	0.995	1.624	0.818
	Jump (n.min-1)	1.98 ± 3.06^b^^,^^c^^,^^d^	0.96 ± 1.26^a^^,^^d^	0.64 ± 0.81^a^	0.37 ± 0.42^a^^,^^b^	< 0.001	< 0.001	< 0.001	0.062	0.001	0.116	0.441	0.605	0.743	0.303	0.629	0.419
	Changes of direction (n.min-1)	5.16 ± 4.38^b^^,^^c^^,^^d^	1.9 ± 1.78^a^^,^^c^^,^^d^	1.23 ± 0.91^a^^,^^b^	0.76 ± 0.51^a^^,^^b^	< 0.001	< 0.001	< 0.001	0.010	< 0.001	0.075	0.965	1.232	1.397	0.477	0.868	0.630
Female	Distance (m.min-1)	198.06 ± 34.5^b^^,^^c^^,^^d^	145.36 ± 22.98^a^^,^^c^^,^^d^	119.19 ± 20.59^a^^,^^b^^,^^d^	97.11 ± 19.02^a^^,^^b^^,^^c^	< 0.001	< 0.001	< 0.001	< 0.001	< 0.001	< 0.001	1.799	2.778	3.626	1.199	2.287	1.114
	Accelerations (n.min-1)	9.72 ± 3.48^b^^,^^c^^,^^d^	4.68 ± 1.86^a^^,^^c^^,^^d^	2.87 ± 1.13^a^^,^^b^^,^^d^	1.96 ± 0.66^a^^,^^b^^,^^c^	< 0.001	< 0.001	< 0.001	< 0.001	< 0.001	0.004	1.819	2.668	3.120	1.174	1.940	0.975
	Accelerations (m.min-1)	24.96 ± 9.24^b^^,^^c^^,^^d^	10.88 ± 4.3^a^^,^^c^^,^^d^	6.13 ± 2.55^a^^,^^b^^,^^d^	4.11 ± 1.39^a^^,^^b^^,^^c^	< 0.001	< 0.001	< 0.001	< 0.001	< 0.001	0.012	1.962	2.788	3.168	1.345	2.121	0.982
	Decelerations (n.min-1)	9.12 ± 3.42^b^^,^^c^^,^^d^	4.48 ± 1.68^a^^,^^c^^,^^d^	2.99 ± 1.13^a^^,^^b^^,^^d^	2.12 ± 0.8^a^^,^^b^^,^^c^	< 0.001	< 0.001	< 0.001	< 0.001	< 0.001	0.005	1.722	2.405	2.817	1.036	1.788	0.890
	Decelerations (m.min-1)	26.7 ± 10.74^b^^,^^c^^,^^d^	11.42 ± 4.3^a^^,^^c^^,^^d^	7.16 ± 2.95^a^^,^^b^^,^^d^	4.65 ± 1.925^a^^,^^b^^,^^c^	< 0.001	< 0.001	< 0.001	< 0.001	< 0.001	0.005	1.871	2.486	2.863	1.154	2.028	1.006
	Sprint duration (s.min-1)	12.66 ± 7.5^b^^,^^c^^,^^d^	5.1 ± 2.94^a^^,^^c^^,^^d^	2.85 ± 1.87^a^^,^^b^^,^^d^	1.60 ± 1.09^a^^,^^b^^,^^c^	< 0.001	< 0.001	< 0.001	< 0.001	< 0.001	0.040	1.318	1.785	2.052	0.914	1.574	0.811
	Jump (n.min-1)	0.9 ± 2.16^b^^,^^c^^,^^d^	0.32 ± 0.74^a^	0.24 ± 0.43^a^	0.16 ± 0.28^a^	0.001	< 0.001	< 0.001	0.594	0.333	0.665	0.362	0.436	0.490	0.155	0.303	0.212
	Changes of direction (n.min-1)	4.14 ± 3.96^b^^,^^c^^,^^d^	1.64 ± 1.28^a^^,^^d^	1.02 ± 0.74^a^	0.56 ± 0.43^a^^,^^b^	< 0.001	< 0.001	< 0.001	0.053	0.001	0.155	0.861	1.111	1.287	0.597	1.133	0.756

The super-indices indicate that the mixed model reveals the existence of differences (*p* < 0.05) between.

a : 10 s.

b : 30 s.

c : 1 min.

d : 2 min.

Among female players, significant differences in jumps per minute occurred only between the 10-second epoch and the 30-, 60-, and 120-second epochs (0.9 ± 2.16 n·min^−1^ vs. 0.32 ± 0.74 n·min^−1^, 0.24 ± 0.43 n·min^−1^, and 0.16 ± 0.28 n·min^−1^, respectively; *p* = < 0.001). No significant differences were observed between the 60-second epoch and the 30- or 120-second epochs for changes of direction per minute in female players (1.02 ± 0.74 n·min^−1^ vs. 1.64 ± 1.28 n·min^−1^; *p* = 0,053; d = 0.597, and 0.56 ± 0.43 n·min^−1^; *p* = 0.155; d = 0.756; respectively).

Significant differences were observed across various time windows between frontcourt and backcourt for both male and female players (Table 2). For males, a significant difference in the distance covered was detected only during the 60-second epoch, with backcourt covering more distance than frontcourt (127.16 ± 18.21 m vs. 118.48 ± 22.77 m; *p* = 0.006; d = −0.427). Additionally, no significant differences were found in the number of accelerations (1.8 ± 0.58 vs. 1.7 ± 0.59; *p* = 0.285; d = −0.167), number of decelerations (1.76 ± 0.5 vs. 1.68 ± 0.55; *p* = 0.311; d = −0.157) and distance covered during accelerations (5.2 ± 1.65 m vs. 4.96 ± 1.85 m; *p* = 0.373; d = −0.138) over the 10-second epoch. No significant differences in sprint duration, number of jumps, or number of changes of direction were observed across all time epochs (*p* > 0.05).

**Table 2:** Average WCS across playing positions during different time window for male and female teams.

Window	Variable	Window															
		10s				30s				60s				120s			
		Frontcourt	Backcourt	*p*	Effect Size	Frontcourt	Backcourt	*p*	Effect Size	Frontcourt	Backcourt	*p*	Effect Size	Frontcourt	Backcourt	*p*	Effect Size
Male	Distance (m)^c^	33.82 ± 6.18	35.56 ± 7.18	0.109	−0.257	74.52 ± 11.54	77.81 ± 12.29	0.071	−0.276	118.48 ± 22.77	127.16 ± 18.21	0.006	−0.427	190.64 ± 39.47	198.12 ± 39.13	0.163	−0.190
	Accelerations (count)^b^^,^^c^^,^^d^	1.7 ± 0.59	1.8 ± 0.58	0.285	−0.167	2.55 ± 0.89	2.91 ± 0.92	0.012	−0.389	3.26 ± 1.26	4.12 ± 1.17	< 0.001	−0.711	4.66 ± 1.85	5.41 ± 1.74	0.006	−0.418
	Accelerations (m)^b^^,^^c^^,^^d^	4.96 ± 1.85	5.2 ± 1.65	0.373	−0.138	6.37 ± 2.52	7.61 ± 2.30	0.001	−0.516	7.84 ± 3.13	9.78 ± 3.07	< 0.001	−0.629	10.57 ± 4.0	12.98 ± 4.13	< 0.001	−0.592
	Decelerations (count)^b^^,^^c^^,^^d^	1.68 ± 0.55	1.76 ± 0.5	0.311	−0.157	2.53 ± 0.8	2.81 ± 0.89	0.030	−0.336	3.3 ± 1.06	3.95 ± 1.24	< 0.001	−0.559	4.31 ± 1.47	5.44 ± 1.72	< 0.001	−0.701
	Decelerations (m)^a^^,^^b^^,^^c^^,^^d^	4.88 ± 1.7	5.65 ± 1.82	0.005	−0.432	6.53 ± 2.06	8.0 ± 2.39	< 0.001	−0.655	7.92 ± 2.55	9.98 ± 3.47	< 0.001	−0.664	10.16 ± 3.41	13.16 ± 4.28	< 0.001	−0.764
	Sprint duration (s)	2.46 ± 1.26	2.7 ± 1.39	0.294	−0.177	2.98 ± 1.51	3.37 ± 1.96	0.157	−0.219	3.26 ± 1.82	3.68 ± 2.28	0.187	−0.198	3.87 ± 1.8	4.52 ± 2.53	0.062	−0.290
	Jumps (count)	0.38 ± 0.57	0.29 ± 0.46	0.252	0.165	0.45 ± 0.67	0.51 ± 0.7	0.401	−0.094	0.58 ± 0.78	0.68 ± 0.83	0.143	−0.128	0.68 ± 0.78	0.79 ± 0.89	0.146	−0.135
	Changes of direction (count)	0.89 ± 0.8	0.83 ± 0.68	0.599	0.082	0.95 ± 0.9	0.96 ± 0.90	0.932	−0.013	1.11 ± 0.92	1.33 ± 0.89	0.145	−0.242	1.43 ± 1.15	1.61 ± 0.93	0.272	−0.173
Female	Distance (m)	32.97 ± 5.93	33.06 ± 5.56	0.933	−0.016	72.96 ± 9.95	72.27 ± 13.54	0.691	0.060	118.99 ± 20.66	119.48 ± 20.8	0.948	−0.024	195.35 ± 42.02	192.63 ± 32.07	0.626	0.071
	Accelerations (count)	1.58 ± 0.61	1.69 ± 0.53	0.309	−0.183	2.28 ± 1.01	2.43 ± 0.81	0.472	−0.159	2.84 ± 1.18	2.91 ± 1.07	0.762	−0.065	3.94 ± 1.32	3.91 ± 1.38	0.858	0.019
	Accelerations (m)^a^	3.77 ± 1.47	4.72 ± 1.47	0.002	−0.644	5.13 ± 2.21	5.88 ± 2.0	0.110	−0.352	5.79 ± 2.67	6.62 ± 2.33	0.139	−0.327	7.75 ± 2.67	8.9 ± 2.83	0.061	0.422
	Decelerations (count)	1.54 ± 0.58	1.49 ± 0.56	0.668	0.095	2.14 ± 0.88	2.37 ± 0.77	0.211	−0.276	2.94 ± 1.28	3.06 ± 0.87	0.639	−0.103	4.06 ± 1.66	4.49 ± 1.5	0.243	−0.267
	Decelerations (m)^a^^,^^b^^,^^d^	3.93 ± 1.62	5.2 ± 1.77	< 0.001	−0.757	5.15 ± 2.19	6.51 ± 1.85	0.002	−0.662	6.68 ± 3.33	7.84 ± 2.17	0.070	−0.397	8.58 ± 4.14	10.33 ± 3.18	0.044	−0.462
	Sprint duration (s)^a^^,^^d^	1.87 ± 1.33	2.45 ± 1.06	0.031	−0.471	2.3 ± 1.44	2.92 ± 1.47	0.054	−0.429	2.58 ± 1.88	3.24 ± 1.81	0.132	−0.355	2.73 ± 2.13	3.9 ± 2.12	0.019	−0.551
	Jumps (count)	0.16 ± 0.37	0.14 ± 0.36	0.827	0.047	0.18 ± 0.39	0.14 ± 0.36	0.656	0.099	0.26 ± 0.44	0.2 ± 0.41	0.426	0.140	0.4 ± 0.61	0.2 ± 0.47	0.086	0.360
	Changes of direction (count)	0.8 ± 0.64	0.54 ± 0.66	0.069	0.398	0.86 ± 0.64	0.77 ± 0.65	0.531	0.138	1.06 ± 0.65	0.97 ± 0.86	0.589	0.119	1.04 ± 0.83	1.26 ± 0.92	0.246	−0.250

The super-indices indicate that the mixed model reveals the existence of differences (*p* < 0.05) between the frontcourt and backcourt, for the windows.

a : 10 s.

b : 30 s.

c : 1 min.

d : 2 min.

For female players, significant differences in distance covered during accelerations appeared only in the 10-second epoch, with backcourt covering more distance than frontcourt (4.72 ± 1.47 m vs. 3.77 ± 1.47 m; *p* = 0.002; d = −0.644). Significant differences in deceleration distance were noted over the 10-second (5.2 ± 1.77 m vs. 3.93 ± 1.62 m; *p* = < 0.001; d = −0.757), 30-second (6.51 ± 1.85 m vs. 5.15 ± 2.19 m; *p* = 0.002; d = −0.662), and 120-second epochs (10.33 ± 3.18 m vs. 8.58 ± 4.14 m; *p* = 0.044; d = −0.462), with backcourt again registering higher values. Additionally, significant differences in sprint duration were detected during the 10-second (2.45 ± 1.06 s vs. 1.87 ± 1.33 s; *p* = 0.031; d = 0.471) and 120-second epochs (3.9 ± 2.12 s vs. 2.73 ± 2.13 s; *p* = 0.019; d = −0.551). No significant positional differences emerged in total distance covered, number of accelerations, number of decelerations, number of jumps, or changes of direction across all time epochs (*p* > 0.05).

## 4 Discussion

This study is the first to characterize multidimensional WCS in elite youth basketball using a composite external-load approach. Our findings highlight significant differences across time windows and playing positions in several physical demand variables.

### 4.1 Effect of time window on worst-case scenarios

The primary finding of this study was that the magnitude of WCS was strongly influenced by the duration of the rolling-average window. Across both male and female players, shorter time windows consistently produced greater relative physical demands than longer time windows for most variables. These findings are consistent with previous investigations in basketball and other intermittent team sports, where shorter epochs have repeatedly been shown to capture more intense passages of play than longer averaging periods (4, 5, 15, 27).

This pattern is likely explained by the mathematical and physiological characteristics of rolling-average analyses. Short windows are more sensitive to brief bursts of intense activity, whereas longer windows inevitably incorporate periods of lower-intensity movement, resulting in a dilution of peak values. Consequently, the selection of the time window substantially influences the magnitude of the physical demands identified and should be carefully considered when interpreting or comparing results across studies.

Interestingly, some variables exhibited a different behavior than locomotor metrics. In both male and female players, jumps and changes of direction showed fewer significant differences between time windows than distance-, acceleration-, and deceleration-based variables. These findings suggest that explosive basketball-specific actions may be less sensitive to the averaging effect induced by increasing window durations. In female players, for example, jump frequency differed primarily between the 10-s epoch and the longer time windows, whereas no significant differences were observed among the longer epochs. Unlike locomotor variables, which accumulate continuously throughout play, jumps and changes of direction are discrete actions generally associated with specific game events such as rebounding, shot contests, defensive rotations, or cutting actions (28). Consequently, their occurrence may depend more on tactical and situational constraints than on the duration of the analyzed time window, which could explain the reduced influence of epoch duration observed in the present study. From a practical perspective, these findings suggest that explosive basketball-specific actions should be incorporated across drills of varying durations, including longer conditioning exercises, to adequately prepare players for the multidimensional demands encountered during competition.

Another notable finding was that the physical demands observed during WCS were consistently lower than the isolated PD previously reported in the same cohort (4). This discrepancy is likely explained by the conceptual differences between both approaches. While traditional PD analyses identify the highest value achieved for a single variable in isolation, the WCS approach implemented in the present study identifies the period characterized by the greatest simultaneous accumulation of multiple external load demands. Consequently, the selected passages do not necessarily correspond to the absolute maximum value of any individual variable but rather to the period where several demands are concurrently elevated. This distinction supports the notion that WCS should not be interpreted as isolated moments of maximal intensity but instead as periods characterized by a high density of concurrent physical demands.

### 4.2 Positional differences during WCS

The present findings indicate that the physical demands experienced during WCS are influenced by playing position, although the magnitude and consistency of these differences varied between male and female players. Overall, backcourt players tended to exhibit greater physical demands than frontcourt players across most of the significant comparisons, supporting previous research demonstrating position-specific differences in basketball match-play demands (4, 16, 28–30).

In male players, backcourt athletes generally displayed greater locomotor demands, particularly for distance covered and acceleration-related variables. These findings are consistent with the tactical and technical responsibilities typically associated with perimeter positions. Guards are frequently involved in ball progression, offensive creation, defensive pressure, and repeated transitions between offensive and defensive phases, all of which require extensive movement and frequent changes in pace (31). In contrast, frontcourt players often perform a greater proportion of their actions in restricted court areas near the basket, where physical performance is characterized by positioning, screening, rebounding, and contact-based actions rather than continuous locomotor activity (29, 30).

Anthropometric characteristics may further contribute to these differences. Frontcourt players generally possess greater body mass and stature than backcourt players (32), potentially limiting their ability to repeatedly perform high-frequency accelerative and decelerative actions during the most demanding passages of play. Conversely, the lower body mass and greater relative mobility of guards may facilitate the accumulation of external load demands across multiple locomotor variables, increasing their likelihood of generating higher composite WCS scores.

Interestingly, positional differences were less pronounced in female players. While backcourt players still demonstrated higher values for several acceleration-, deceleration-, and sprint-related variables, the number of significant differences was substantially lower than in males. These findings align with previous studies reporting smaller positional disparities in female basketball compared with male basketball (4, 33, 34). Scanlan et al. (34), for example, observed differences in specific activity patterns between positions but reported a less distinct positional specialization than that typically observed in men's basketball. Similarly, Delextrat and Cohen (33) suggested that although positional differences exist in physical qualities such as agility and anaerobic performance, these distinctions may be less pronounced among female athletes.

An additional and potentially important observation is that positional differences appeared less evident when using a multidimensional WCS approach than when peak demands are examined independently. Previous investigations based on isolated PD variables have consistently reported higher values for backcourt players across numerous metrics (4). However, the present findings suggest that when several external load variables are simultaneously considered within a single composite scenario, the differences between positions become less pronounced. This may indicate that although players reach position-specific maxima through different physical strategies, the overall accumulation of external load during the most demanding passages of competition is more comparable than previously assumed.

### 4.3 Study limitations

Although the present findings provide novel insights into WCS in elite youth basketball, several limitations should be acknowledged.

First, the current approach was exclusively based on external load variables. Internal load responses (e.g., heart rate, perceived exertion, or physiological strain) and contextual factors such as score margin, opponent strength, game quarter, tactical strategies, game phase, or home-court advantage were not considered. Given the multidimensional nature of basketball performance, these factors may substantially influence the demands experienced by players during the most challenging passages of play (35).

Second, all external load variables contributed equally to the composite WCS score. Although this decision avoided introducing subjective weighting assumptions, it remains possible that certain physical actions may contribute more substantially than others to overall match demands. Future studies should explore alternative approaches, including weighted models, multivariate dimensionality-reduction techniques, or machine-learning methods, to determine whether different weighting strategies improve the characterization of WCS.

Third, the study was conducted exclusively in elite youth basketball players from a single high-performance institution, which may limit the generalizability of the findings to other competitive levels, age categories, or playing standards. In addition, only home matches were included in the present investigation. Although this approach ensured consistency in the tracking environment, it may have introduced a home-court effect, which has previously been reported to influence physical performance and match demands in basketball (36, 37). Consequently, caution is warranted when generalizing these findings to away or neutral-court competitions.

Furthermore, no *a priori* sample size calculation was performed. This study was based on the complete population of elite youth basketball players available within a single high-performance institution, which is characteristic of observational research conducted in highly selective athletic populations. Although this approach maximized the available sample and ensured ecological validity, the relatively limited number of participants may have reduced the statistical power to detect smaller effects and should be considered when interpreting and generalizing the present findings.

Finally, despite incorporating multiple external load variables simultaneously, the proposed WCS framework remains a simplified representation of basketball performance. Technical, tactical, perceptual, and decision-making demands were not included in the present model and may contribute substantially to the complexity of the most challenging moments encountered during competition. Future research should therefore seek to integrate external load with internal load measures (e.g., heart rate and perceived exertion), together with physiological and contextual information, to provide a more comprehensive characterization of basketball WCS.

## 5 Conclusion

This study is the first to investigate WCS in elite youth basketball using a multidimensional approach integrating multiple external load variables simultaneously. The findings demonstrate that the physical demands experienced during WCS are strongly influenced by the duration of the rolling-average window, with shorter epochs producing greater relative demands than longer time windows. In addition, positional differences were observed, particularly among male players, with backcourt players generally exhibiting greater locomotor demands than frontcourt players during the most physically demanding passages of play.

Importantly, the present findings demonstrate that WCS should not be interpreted as isolated moments of maximal intensity but rather as periods characterized by the greatest concurrent accumulation of multiple external load demands. Consequently, the multidimensional nature of WCS provides information that differs from traditional PD analyses and may offer a more comprehensive representation of the physical challenges encountered during basketball competition.

From an applied perspective, these findings support the use of position-specific and time-window-specific conditioning strategies when preparing athletes for competition. More broadly, the proposed framework may assist practitioners in moving beyond isolated performance indicators toward a more holistic understanding of the most demanding passages of play. Future research should seek to integrate physiological and contextual information, as well as alternative weighting strategies, to further refine the multidimensional characterization of worst-case scenarios.

## Data Availability

The original contributions presented in the study are included in the article/Supplementary Material, further inquiries can be directed to the corresponding author.
